# Overview of Anorectal Malformations in Africa

**DOI:** 10.3389/fsurg.2019.00007

**Published:** 2019-03-05

**Authors:** Taiwo A. Lawal

**Affiliations:** Division of Pediatric Surgery, Department of Surgery, University of Ibadan and University College Hospital, Ibadan, Nigeria

**Keywords:** Africa, anorectal malformations, burden of diseases, low resource settings, neonatal intestinal obstruction

## Abstract

Anorectal malformation is one of the most common structural congenital malformations treated by pediatric surgeons globally. The outcome of care is largely dependent on the spectrum, clinical features, associated malformations, expertise of the surgeons, and available perioperative facilities. Africa has a large burden of unmet surgical needs in children, and as in other low resource settings, local pediatric surgeons are faced with different and challenging clinical scenarios, hence, adopt various measures to enable children with surgically correctable congenital malformations to survive. There are increasing collaborations between local surgeons and experts in other continents, which often involves surgeons traveling to Africa on missions or well-structured partnerships. It is highly beneficial for the physician who is interested in global-surgery to understand the terrain in low resource settings and prepare for possible changes in management plan. This review highlights the epidemiology, clinical presentation, treatment, and outcome of care of children with anorectal malformations in Africa and provides options adopted by pediatric surgeons working with limited resources.

## Introduction

Anorectal malformation (ARM) is a spectrum of structural congenital defects involving the anorectum and variable segments of the urogenital system in boys and girls (1). The malformations range from skin level defects such as rectoperineal fistulas to complex lesions such as persistent cloaca. The cause has not been fully elucidated but it is likely to be multifactorial and include genetic and environmental factors (1, 2). The prognosis of ARM is related to the complexity of the malformation. Some of the most complex malformations are not easily treatable by all practitioners since those types occur infrequently and may be best handled by experts who are more familiar with them (3).

The treatment options, often, are influenced by factors related to the clinical presentation and facilities available for the perioperative care of children with complex congenital malformations. The epidemiology, clinical presentation, course, and outcome of care of ARM in Africa may thus be different from what occurs in other regions, hence, this review, which aims to highlight those aspects of management of patients with the malformation on the continent and provide options adopted by pediatric surgeons working with limited resources.

## Epidemiology of Anorectal Malformations in Africa

Congenital malformations account for one third to two fifths of the operative workload of pediatric surgeons in typical major referral hospitals in Africa (4, 5). ARM is the commonest major structural congenital malformation presenting to general pediatric surgeons on the continent (5). ARM is also the leading congenital cause of intestinal obstruction in African children (6, 7). The treatment of children with ARM is a major aspect of the work of pediatric surgeons as colostomy for ARM and the definitive anorectoplasty or anoplasty are the commonest colorectal procedures they perform (3).

A true birth incidence of ARM is difficult to obtain because there are no formal birth registries in most parts of Africa and most reports in the literature are hospital based. The best available population based estimates are from South Africa where the incidence of ARM has been reported to range from 1.79/10,000 live births in the Western Cape Region to 3.26/10,000 live births on the West Coast (8). These figures are about the same as the incidence of 1 in 5,000 live births reported elsewhere (1).

There is a slight to moderate male preponderance in cases of ARM ranging from 55 to 71% according to majority of reports (8–14) but a few had shown the reverse (15–18), which could be due to loss of male neonates from delay in seeking care in the catchment areas of the hospitals concerned. Rectovestibular fistula is the predominant type of ARM seen in females representing 70–78% of the malformations in girls (8, 14, 17, 19–21).

In boys, the spectrum is slightly more varied across the continent. Imperforate anus without fistula was the predominant type seen in 31–42% of boys with ARM in Kenya (14, 22). On the other hand, ARM with rectourethral fistula predominates in boys in Nigeria (9), Cape Town in South Africa (20), Ethiopia (17), Uganda (21), and in Malawi (23). A major limitation encountered in comparing the spectrum of malformations in various publications is the use of different and inconsistent classification schemes by various authors. The adoption of the Krickenbeck consensus classification scheme (24) has resulted in less confusion about the terminologies since the classification is clinically oriented (25).

The presence of associated malformations in other systems is seen in 9–44% of patients in various series across the continent (9, 12, 15, 17–19, 26). Those figures are lower than the expected and more widely documented proportion of 50–78% (27–30) of patients with ARM who have associated malformations. Conversely, in a recent review of 282 children at the Red Cross War Memorial Children's Hospital, Cape Town using a more robust evaluation method, 152 (69%) children were reported to have associated malformations in other systems (20).

The lower incidence of associated malformations in most reports from Africa have been attributed to higher mortality in the neonatal period (9, 26), less accurate detection and possibly because some children with more lethal associated defects would never be seen at a hospital after birth at home and subsequent demise (26). Some patients may be lost to follow-up after initial colostomy including some that may die from consequences of undiagnosed and untreated associated malformations (15). It is therefore, not improbable that the incidence of associated malformations in African children with ARM will increase as neonatal survival improves and many children reach the stage where accurate evaluations can be made.

## Challenges in the Neonatal Period

Early management is recommended in the treatment of children with ARM in order to prevent sepsis and other morbidities related to intestinal obstruction (1). Neonatal intestinal obstruction is a major surgical emergency that requires optimal neonatal resuscitation facilities, surgical care and anesthetic support. Neonatal intestinal obstruction is responsible for 24–64% of neonatal surgical admissions in Africa (31–33). ARM is a major cause accounting for 57–67% of cases of neonatal intestinal obstruction (31, 32, 34).

Most children with ARM and clinically significant intestinal obstruction will require neonatal intensive care unit (NICU) support. This is to ensure optimal control of hypothermia, fluid and electrolyte balance, parenteral nutrition and respiratory support. These NICU facilities are largely unavailable in many centers. A cross-sectional study of pediatric surgeons and surgical capacity in West Africa showed that only 51% of 37 (surveyed) hospitals had a NICU or general ICU facility (35). The situation is more critical in very low birth weight or extremely premature babies with ARM as well as in those with severe associated congenital malformations involving the cardiovascular or respiratory systems. In some situations, pediatric surgeons often resort to dilatation of perineal fistulas in male and female neonates or vestibular fistulas in female neonates with very low birth weights or that are extremely preterm to gain time and improve chances of survival (25, 36). Expansion of neonatal surgical support services has been advocated as a way of improving the outcome of care of children requiring neonatal surgical care (34).

## Delay in Presentation and Treatment

**Figure 1:**
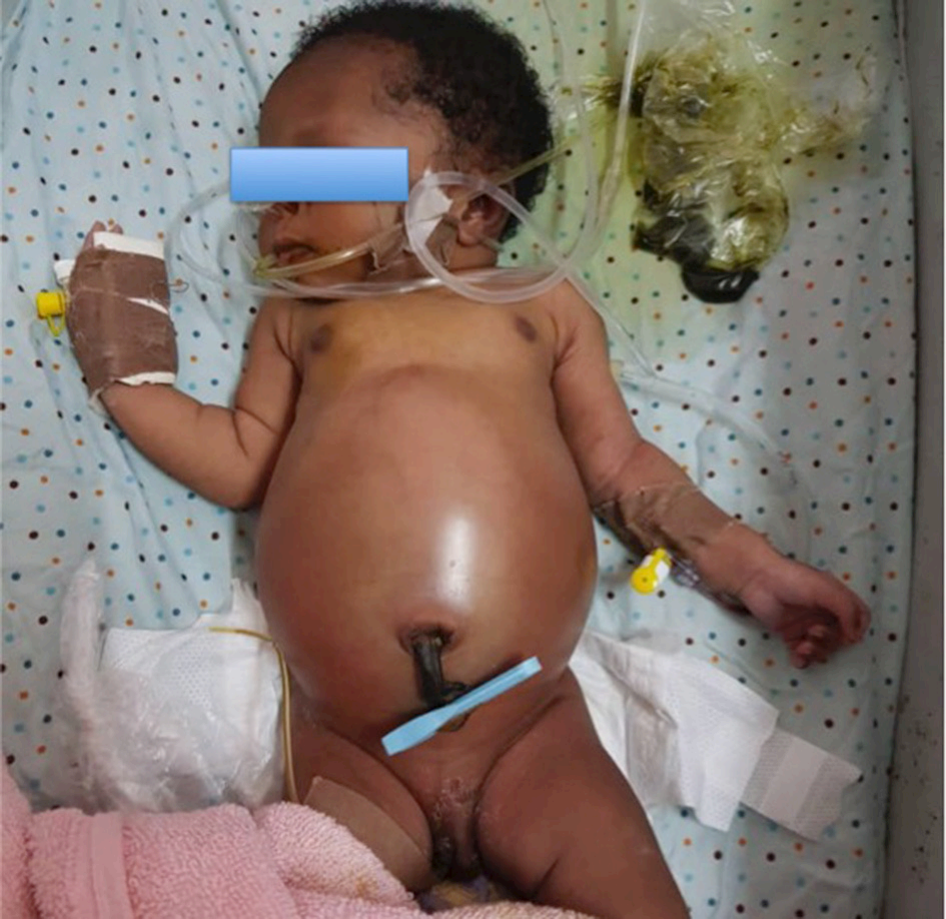
A neonate brought to the hospital on the fourth day of life with intestinal obstruction secondary to anorectal malformation.

**Figure 2:**
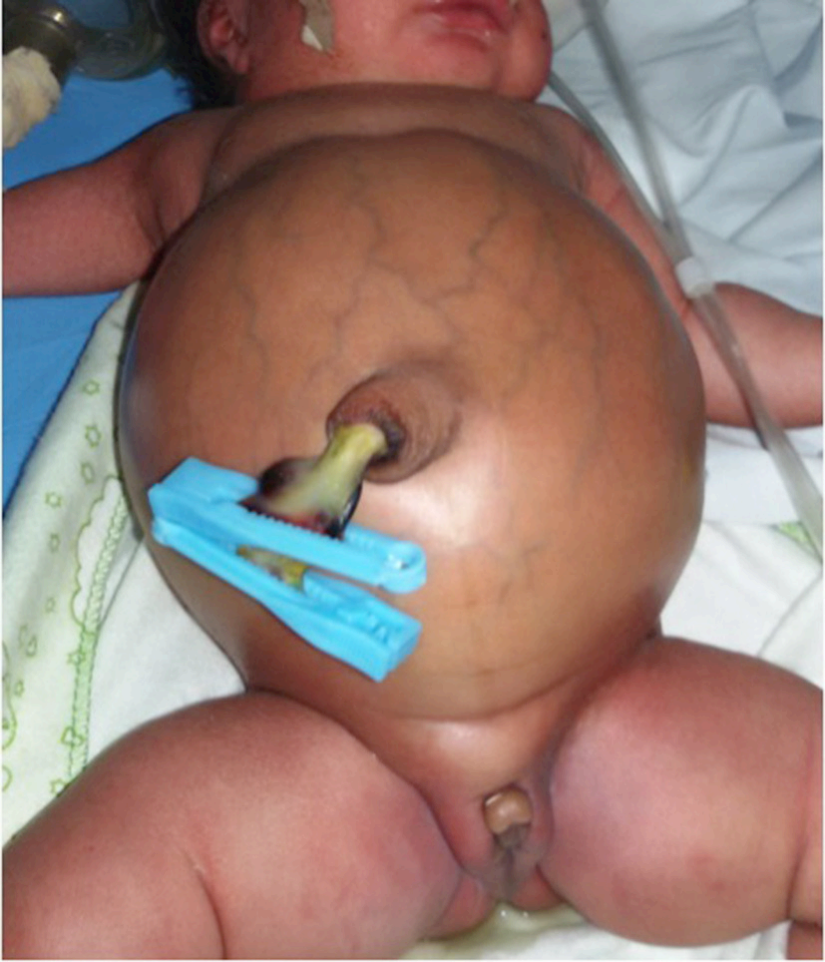
A neonate with cloacal malformation and massive abdominal distension at presentation on the 8 day of life.

Delay in presentation of patients with ARM leads to progression of neonatal intestinal obstruction (Figures 1, 2), sepsis, aspiration pneumonia, intestinal perforation, and sometimes death (13, 17, 37–39). Adejuyigbe et al. (9) in a retrospective review of 86 patients managed on account of ARM at Ile Ife, Nigeria reported that 74 (86.0%) presented after 24 h of birth. In that series, nearly 60% of the patients presented with gross abdominal distension and over one-quarter had associated vomiting (9). Govender and Wiersma (40) in a retrospective study of 273 patients who presented to a tertiary hospital over a period of 8 years reported that 158 (57.9%) presented after 24 h of birth i.e., delayed. Similarly, 63% of 78 children treated for ARM at a major referral center in southwest Uganda presented after 48 h of birth (21).

Delayed presentation is worse among female children because there is still some fecal discharge through the vestibular fistula in most instances unlike in males where abdominal distension occurs over a few days in most patients with a rectourethral fistula (12, 36). Urosepsis with septicemia is also more likely in males and it is possible that some of the boys who are not identified early would have died before reaching health care 8facilities (23).

Reports indicate that a large proportion, ranging from 25 to 94.8% of childbirths in Africa take place outside the hospital environment (4, 41–46). A pediatrician, within a reasonable length of time after delivery, does not get to examine the majority of newborns as a result. Delivery outside a hospital setting is significantly associated with delayed diagnosis of ARM (40). It is sometimes up to the mothers and more experienced family members to identify children with gross structural anomalies (47). This is of course fraught with challenges, as most mothers had never heard of ARM (48). Furthermore, one out of every five mother, from a study conducted in Ibadan, Nigeria, was able to recognize babies with ARM (48). Delayed presentation has been largely attributed to delay in detection at birth, erroneous information given to parents, poor knowledge of birth defects, suboptimal treatment and socioeconomic factors (12, 49).

## Presentation Outside the Neonatal Period

Presentation outside the neonatal period is widespread as 19–85% of patients with ARM in Africa present outside the first 4 weeks of life (3, 9, 12, 14, 15). Delay in diagnosis has been correlated with poorer outcome and a higher mortality (9).

Patients with ARM sometimes present outside the neonatal period in other regions as well, but to a much less extent (50, 51). Delayed diagnosis beyond 24–48 h of birth is unusual in developed countries because of adherence to guidelines on newborn care since most deliveries are supervised. There may also be delay in diagnosis when the malformation is subtle or a rare variant such as H-type fistula is encountered (52, 53). Furthermore, extreme delays beyond childhood are uncommon except in low resource settings such as in many developing countries.

It is not unusual to see patients present well outside infancy in Africa (12, 54). Ogundoyin et al. (54) reported a 25-year-old Nigerian woman with ARM and rectovestibular fistula who then underwent a successful repair after an initial colostomy. Other authors have reported series with patients well outside of infancy and sometimes in adolescence (9, 12, 55). Furthermore, when patients present early enough for treatment, there are tertiary level delays in intervention and the median time to emergency surgery may be as high as seven days as reported in a study that reviewed the pediatric surgical capacity in Africa (56). This delay is as a result of inadequate systemic support for pediatric surgical services, which often requires a multidisciplinary form of care and is human capita intensive (56, 57).

## Surgical and Perioperative Considerations

The perioperative care of patients with ARM typifies the advancement of multidisciplinary care. Multidisciplinary approach has been shown to result in more optimal care of patients with ARM (58). Outside of Northern and Southern Africa and a few countries in Western Africa, many hospitals do not have a good complement of personnel to form multidisciplinary teams and the pediatric surgeon has to work with minimal resources and perform multiple roles, including training other staffs, to ensure that children with surgical conditions are given a chance to survive (57, 59–61).

There is a wide variation in the timing of the definitive procedure based on a multiplicity of factors that are general, i.e., globally relevant, and those that are specific to the region. General factors include the type of malformation, associated congenital malformations, expertise of the surgeon and availability of related specialists. Factors that are specific to the timing of definitive surgery on the continent include economic considerations for funding of surgery, imaging facilities, availability of experts skilled in neonatal anesthesia, and support facilities such as parenteral nutrition and neonatal intensive care (13, 14, 23, 62).

Table 1 illustrates the timing of definitive surgery for ARM in reports across Africa.

**Table 1:** Timing and technique of definitive surgery for anorectal malformations in available publications across Africa.

**Publication**	**Country**	**Technique for “low” ARM**	**Time of repair of “low” ARM**	**Technique for “higher” ARM**	**Time of repair of “higher” ARM**
Shija (63)	Zimbabwe	Cutback or transfer anoplasty	At presentation	Abdominoperineal pull-through	1–2 years of age
Abdalla et al. (64)	Egypt			PSARP	Age: 5 months to 8 years (mean−23 months). 2 months to 3 years after colostomy (mean−9 months).
Kigo and Ndung'u (22)	Kenya	PSARP	Not indicated	PSARP	10.9% had definitive surgery by age of 6 months; 90.5% waited for over 6 months after colostomy
Archibong and Idika (15)	Nigeria	Cut-back anoplasty	Not indicated	Abdominosacroperineal pull-through	Not indicated
Adejuyigbe et al. (9)	Nigeria	Anoplasty	Neonatal period	Sacroperineal pull-through & PSARP.	4–12 months after colostomy
Ntia et al. (65)	Nigeria	Cut-back anoplasty, perineal transplant	Neonatal period	PSARP	Not indicated
Beudeker et al. (23)	Malawi	Anoplasty	Neonatal period	PSARP	Not indicated
Kuradusenge et al. (14)	Kenya	Anoplasty	Neonatal period	PSARP & ASARP	Mean of 211 ± 111 days
Gama and Tadese (17)	Ethiopia	Anoplasty	Neonatal period	PSARP & ASARP	Not indicated
Kayima et al. (21)	Uganda			PSARP	Median age of 11 months
Mfinanga et al. (18)	Tanzania	Anoplasty	Neonatal period	PSARP	Not indicated

*ARM, anorectal malformation; ASARP, anterior sagittal anorectoplasty; PSARP, posterior sagittal anorectoplasty; NB, Studies focused on selected subset of patients were excluded*.

In view of the aforementioned challenges, most patients with ARM, other than rectoperineal fistulas, will have a diverting colostomy at presentation. The initial colostomy is life saving and done as an emergency procedure; it is not unusual that this procedure may be performed by a non-pediatric surgeon or non-surgeon (36). It is important that most physicians practicing in the continent, especially in remote rural areas, are able to perform colostomy in neonates (41).

**Figure 3:**
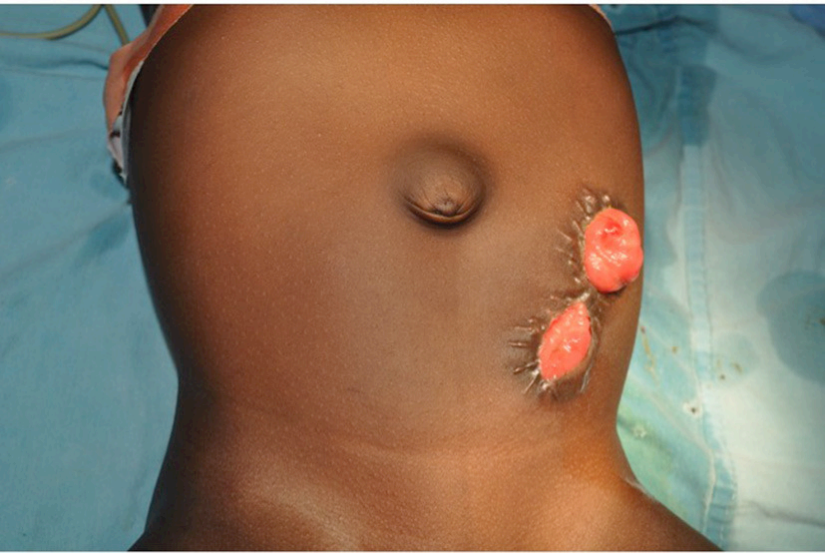
A divided distal descending/sigmoid colostomy in a girl with anorectal malformation.

The colostomy of choice in the treatment of children with ARM is a divided distal descending/sigmoid colostomy (Figure 3) (66). Transverse colostomy, although faster and easier to construct, is generally not optimal except in extremely few patients with cloaca malformations that may benefit from vaginal replacement with the sigmoid colon (66). It was popular when there were relatively very few pediatric surgeons thus general practitioners and general surgeons constructed most colostomies (22). Transverse colostomy is less favored in the management of children with ARM because of the long length and surface of colon that could be in contact with the urinary tract through the fistula. It is also more difficult to clean out, it makes the high-pressure distal colostogram quite challenging to perform or interpret and has a high tendency to prolapse (36, 66, 67). On the other hand, a divided distal descending/sigmoid colostomy ensures adequate fecal diversion and provides a better opportunity to do a high-pressure distal colostogram.

A divided distal descending/sigmoid colostomy is increasingly being performed for fecal diversion in the management of patients with ARM (67–69). An historical comparison of the management of children with ARM over two decades in Zaria, Nigeria showed that 69 patients who required a colostomy in the first 10 years (January 1988 to December 1997) of the review had transverse loop colostomy compared to 96 patients in the latter 10 years (January 1998 to December 2007) who had divided sigmoid colostomy (13). Loop colostomies are also not advisable in patients with ARM, because they may allow spillage into the distal limb with the possibility of recurrent urinary tract infections (66) and they are more likely to prolapse (70). A persistent concern though is the inadequate number of pediatric surgeons on the continent, especially in rural towns (59, 61), with 0.06–2.6 pediatric surgeons per million population under 14 years (57, 60, 61). The result is that there remains a high unmet surgical need in children especially in sub-Saharan Africa (71–73).

Colostomy for ARM may be associated with complications in 12.9–78.9% (Table 2) of African children (14, 18, 68, 69, 74). The most commonly reported complications following colostomy for ARM are prolapse, skin excoriations and hemorrhage (Table 2). Colostomy prolapse may occur because of the use of highly mobile segment of the colon such as the transverse colon or siting a loop sigmoid colostomy rather than the recommended divided distal descending/sigmoid colostomy (36, 66, 67). Skin excoriations are rather much commoner than seen elsewhere (66, 75, 76). This may not be unconnected with the non-availability or non-affordability of appropriate stoma bags for children in some countries (69, 74, 77). Local surgeons have adapted the use of pieces of absorbent clothing material or improvised bags to collect the feces (69, 78). The use of such materials allow feces to stay in contact with the skin for long periods of time, which provides a milieu for maceration of the skin, infection and enzymatic auto-digestion of the macerated skin (77). Zinc oxide powder and/or petroleum jelly based ointments have been applied on the skin that will be in contact with feces to reduce the occurrence as well as to treat this complication (74, 77). The reported mortality rate after colostomy for ARM range from 0 to 25.4% (Table 2) (9, 18, 68, 69, 74).

**Table 2:** Common complications following colostomy for patients with anorectal malformations.

**Publication**	**Number who had colostomy**	**Number with complications (%)**	**Prolapse**	**Bleeding**	**Skin excoriation**	**Mortality no (%)**
Adejuyigbe et al. (9)	59	NA	NA	NA	NA	15 (25.4)
Chirdan et al. (68)	61	16^*^ (26.2)	3	2	NA	12 (19.7)
Lukong et al. (69)	38	7 (18.4)	0	0	2	2 (5.3)
Kuradusenge et al. (14)	31	4 (12.9)	1	0	NA	NA
Aiwanlehi et al. (74)	19	15 (78.9)	4	3	15	0 (0)
Mfinanga et al. (18)	107	34 (31.8)	11	5	8	8 (7.5)

*NA, information not available in the publication*.

* *12 patients had superficial surgical site infections. NB, Studies limited to patients who had colostomy for anorectal malformations*.

Anesthesia in the neonatal period is necessary for the procedures of diverting colostomy for rectourethral fistulas in boys, rectovestibular fistulas and cloaca malformations in girls as well as definitive anoplasty for rectoperineal fistulas. This is however, not always available. In some hospitals, non-physician anesthetic personnel are involved in anesthetizing children and are limited in the scope of their work (79). There are safety issues involved since these personnel are not fully trained in neonatal physiology and pharmacology necessary to have a full grasp of the surgical and anesthetic concerns of a surgical neonate. Where facilities for general anesthesia in children are available, inadequate support services such as monitoring under anesthesia has been shown to lead to poorer outcomes in infants, children undergoing emergency surgery and American Society of Anesthesiologists (ASA) score of three or higher (80). Anesthetic considerations are quite important considering that 44% of emergency neonatal surgical interventions are as a result of ARM (34).

General anesthesia for neonates may not be available, safe or feasible and in some instances, local anesthesia is used (68, 69) but this is less than optimal because of the difficulty with examination of the peritoneal cavity, washing out of the distal colon and lack of abdominal wall relaxation. In addition to the availability of personnel and equipment, another factor that may determine the type of anesthetic used is the infant's general condition at operation; with low birth weight, premature or unstable infants more likely to undergo procedures under local anesthesia. In a comparison of two groups of neonates (one with weight < 2.5 kg and the other of weight >2.5 kg at presentation) who had colostomy for ARM, 18/23 (78.3%) in the first group compared to 20/38 (52.6%) in the second group had the surgery under local anesthesia (68). Where neonatal intensive care facilities or support services are absent, local anesthesia combined with oxygen supplementation via nasal catheter has helped to lower the mortality and will likely continue to be used in some children in low resource settings (68, 69, 81). In some countries, there has been improvement in anesthetic facilities for neonates and an increasing number of neonates are offered general anesthesia even by non-physician anesthetists, especially nurse anesthetists (34, 82).

**Figure 4:**
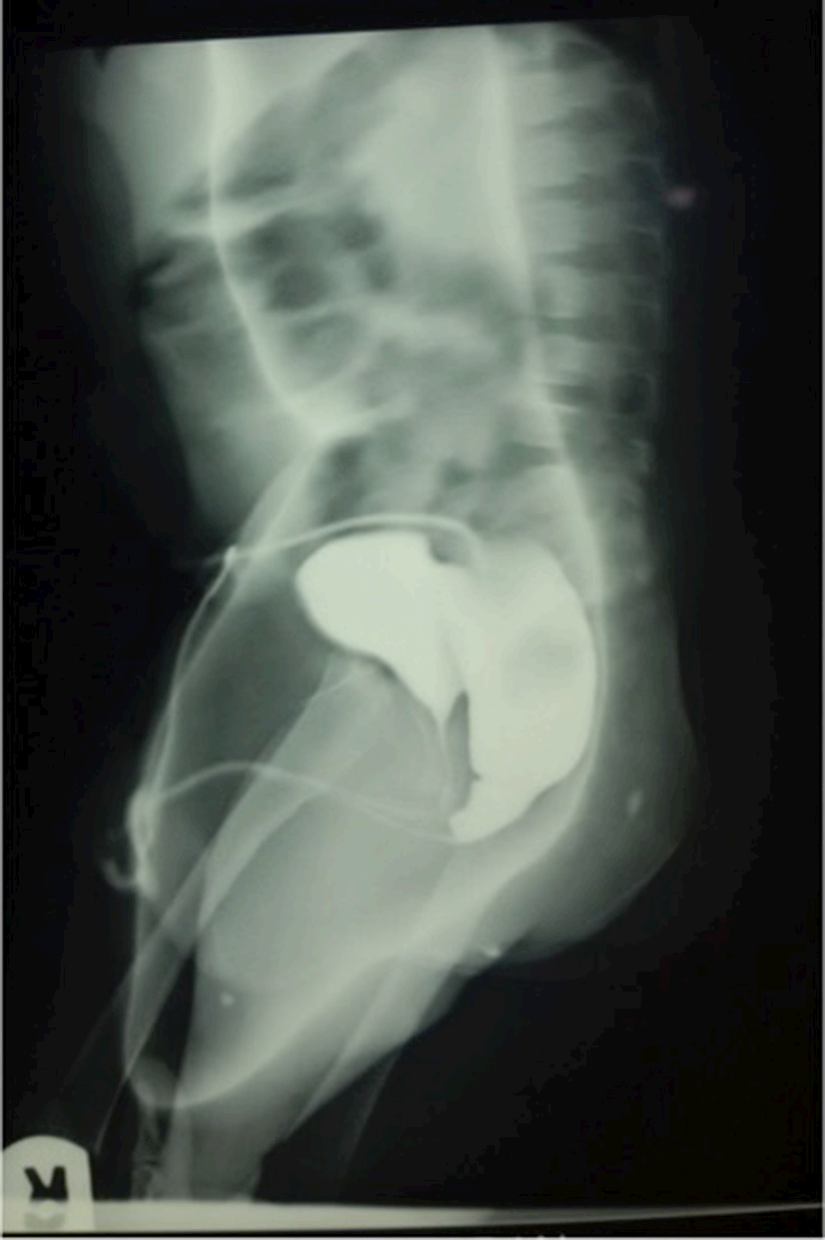
A high-pressure distal colostogram in a male infant with rectobulbar urethral fistula.

Imaging support is crucial in the care of patients with ARM (83). This includes abdominal and pelvic ultrasound scans to evaluate associated urinary and reproductive tract malformations shortly after birth, cross table lateral radiograph to classify the malformation and a high pressure distal colostogram (Figure 4) to define the specific type of rectourethral fistula in boys (1, 25, 83, 84). Others include radiographs to prognosticate the condition and magnetic resonance imaging (and ultrasound scan) to define the anatomy, screen for tethered cord and evaluate for other associated malformations (25, 83–86). While basic imaging is available in many centers, many patients will not benefit from some important evaluations because of absence of facilities, lack of radiologist, lack of fluoroscopy guidance or economic considerations (87). The more widespread nature of ultrasonographic facilities has been suggested as a way forward in regions with limited resources (36). Perineal ultrasound can be used as an adjunct to estimate the distance between the rectal pouch and the skin (36, 87).

At many of the pediatric surgical centers in Africa, definitive surgery for most malformations, other than perineal fistulas, is done outside the neonatal period (67, 88). More recently, some centers are performing primary definitive surgery for ARM in the neonatal period. Osifo et al. (89, 90) performed a primary Posterior Sagittal Anorectoplasty (PSARP) in five well-selected patients with rectovestibular fistula, rectourethral fistula and ARM without fistula. All five patients were operated between the second and seventh days of life and were discharged home between eight and ten days after surgery. Elhalaby in Egypt (91) and Adeniran et al. (92) in Ilorin, Nigeria had also demonstrated the feasibility of primary PSARP without colostomy in carefully well-selected males with “intermediate” types of malformations. Others have also performed primary PSARP for girls with rectovestibular fistulas (13, 91, 93). It is to be noted, however that experienced surgeons practicing in centers with good neonatal perioperative support did those and the rate of wound infection, which may be as high as 26.3% (94) may be quite a challenge to manage in most hospitals (91). Thus, most pediatric surgeons, even in countries with good support facilities, will recommend staged approach for boys with ARM apart from those with perineal fistulas (67, 88).

The most commonly adopted technique for perineal fistula is anoplasty and for “higher lesions” is a PSARP (Table 1). The PSARP technique, as popularized by Peña and Devries (95) is easy to teach and adopt and follows anatomical landmarks. The procedure involves identification of the distal rectum, separation and ligation of any fistula, mobilization of the rectum, and placing it within the muscle complex and parasagittal fibers of the external sphincter (95). There is a need for intraoperative muscle stimulation to identify where to place the distal rectum. Improper localization may lead to fecal incontinence and the need for a reoperation. While a few centers have the Peña muscle stimulator for this purpose (64), most hospitals lack this facility and therefore improvise with various forms of nerve stimulators (36, 96). Others use regular electrocautery but this is more likely to be successful if the sphincter muscles are fairly well-developed (36). Other approaches to definitive surgery include the anterior sagittal anorectoplasty (ASARP), which aims to preserve the internal sphincter and is sometimes used for females with rectovestibular fistulas (14, 17). Sacroperineal and abdomino-sacroperineal pull-throughs were practiced in the past (9, 15) but not reported in recent series (Table 1). Laparoscopy assisted anorectal pull-through (LAARP) is feasible, but is largely limited to Egypt and South Africa (96, 97).

## Special Considerations in Management

The prognosis following management of patients with ARM on the continent mirror what is reported for the various malformations in the literature (1). A major challenge though remains continuity of care as only a few series had reported excellent long-term follow up periods. The reasons for this may be multifactorial and include accessibility to subsequent care, nomadic population and migration, parents not wanting the child because of erroneous beliefs about the malformation etc. (9, 15, 71, 98).

Rectovesical fistulas in boys are associated with the worst prognosis for bowel control while perineal fistulas have the best prognosis (1, 9, 18, 22, 90). In a study evaluating long term outcome in patients who had surgery for ARM in Kenya, voluntary bowel movement was reported among 79.1% of boys and 75% of girls with perineal fistula, 76% of girls with vestibular fistula, 73.9% of boys with rectourethral fistula and 12.5% of boys with rectovesical fistula (22). Similarly, 33 of 37 patients (89.2%) available for long-term follow-up in Ile Ife, Nigeria had “good outcome”; with occasional soiling in four and incontinence in one patient (9). Others also reported similar outcome with “good bowel motion” or fecal continence in 62.5–90.1% (21, 64, 65) following surgery for children with ARM. In addition, prognosis for continence tends to be better in children who had surgical interventions earlier than later (18, 22).

Following definitive surgery, complications are reported among one third of the patients (Table 3) although actual figures may be higher in some series as patients may be lost to follow up and some of those may die (15). The most prevalent complications (Table 3) are wound infection and or sepsis with or without wound dehiscence (10.4%), anal strictures (8.6%), and prolapse (5.3%). Septic complications can occur after surgery for “low,” “intermediate,” or “high” malformations. Staged approach, for all malformations, other than perineal fistulas, is highly recommended to reduce septic complications (9, 11, 36, 91).

**Table 3:** Complications following definitive surgery for patients with anorectal malformations in published series from Africa.

**Publication**	**Number of patients**	**Prolapse**	**Anal stricture**	**Wound dehiscence/infection/sepsis**	**Other complications**
Abdalla et al. (64)	51	0	2	4	3^@^
Archibong and Idika (15)	54	NA	6	NA	7^@^
Adejuyigbe et al. (9)	42^*^	5	5	6	4
Elhalaby (91)^**^	38	0	5	9	11^#^
Makanga et al. (11)	46	NA	2	10	8^***^
Ntia et al. (65)	53^*^	5	3	0	3^@^
Elbatarny et al. (94)^##^	38	5	4	10	1
DeVos et al. (97)^**^	73	6	4	6	0
Beudeker et al. (23)	46	2	6	5	1
Osagie et al. (90)^α^	33	2	3	3	0
Abdelmaksoud et al. (96)^β^	20	8	0	1	5 ^ββ^
Gama and Tadese (17)	96^*^	NA	12	4	10
Mfinanga et al. (18)	36^*^	0	2	7	10^****^
Total No (%)	626	33 (5.3)	54 (8.6)	65 (10.4)	63 ^βββ^

*NA, information not available in the publication*.

@ *Fecal incontinence*.

* *These were the patients who had definitive surgery among the patients reported in the series*.

** *Patients in this report had high or intermediate malformations*.

# *Six patients had injury to the posterior vaginal wall*.

*** *Six patients died*.

## *Single stage approach used*.

α *Patients had high malformations only*.

β *Study limited to laparoscopy assisted anorectal pull-through in boys*.

ββ *Three patients re-operated for retraction and mislocation*.

**** *Seven patients died*.

βββ *Highly variable as some series did not comment on incontinence as a complication and some did not report mortality rate after definitive surgery*.

Skin level anal stricture, is a major preventable complication after definitive surgery, and has been reported in 5–14% of patients (9, 13, 15, 90, 91). This complication was seen after surgery in as much as 49% of patients before the adoption of PSARP as the approach for definitive surgery (13). Skin level anal stricture is due to failure of compliance with anal dilation regimen while deeper and more fibrotic stricture occurs because of vascular injury during handling and mobilization of the distal rectal stump (99). The majority of cases of anal strictures are amenable to anal dilatation. An option that has been adopted is to make the initial anoplasty wider than usual in older patients with larger rectal pouches and those that are likely difficult to follow up in the hospital (36).

The overall mortality in children with ARM range from 4.3 to 31.0% (9, 14, 18, 21, 63, 65). The mortality rate is higher in children with associated malformations (9, 14), those with “higher” malformations (11) and in the neonatal period (9). The commonest causes of death are associated malformations and sepsis (9, 14).

## Conclusion

Anorectal malformations account for a major part of the workload of pediatric surgeons practicing in Africa; the epidemiology, clinical features and preoperative work-up are quite varied but delayed presentation is usual, especially in sub-Saharan Africa. A huge challenge in management is the inadequate number of pediatric surgeons or support services and facilities to care for these children especially in the neonatal period. The outcome of surgery is dependent on the specific type of malformation but is better when intervention is commenced early. Early diagnosis, improvement in neonatal intensive care support, especially for children with associated malformations in other systems, and prevention of sepsis will help reduce the mortality rate.

## Author Contributions

The author confirms being the sole contributor of this work and has approved it for publication.

### Conflict of Interest Statement

The author declares that the research was conducted in the absence of any commercial or financial relationships that could be construed as a potential conflict of interest.
